# When nanomedicines meet tropical diseases

**DOI:** 10.3762/bjnano.15.69

**Published:** 2024-07-08

**Authors:** Eder Lilia Romero, Katrien Van Bocxlaer, Fabio Rocha Formiga

**Affiliations:** 1 Nanomedicine Research and Development Centre (NARD), Science and Technology Department, National University of Quilmes, Roque Saenz Peña 352, B1876 Bernal, Provincia de Buenos Aires, Argentina; 2 Skin Research Centre, Hull York Medical School, York Biomedical Research Institute, University of York, York YO10 5DD, UK; 3 Aggeu Magalhães Institute, Oswaldo Cruz Foundation (FIOCRUZ), 50670-420, Recife, PE, Brazil; 4 Faculty of Medical Sciences, University of Pernambuco (UPE), 52171-011, Recife, PE, Brazil

**Keywords:** *Aedes aegypti*, Chagas disease, leishmaniasis, nanomedicine, nanotechnology, neglected tropical diseases, schistosomiasis

In May 2021, the World Health Assembly from the World Health Organization (WHO) decided to officially recognize January 30th as the World Neglected Tropical Diseases Day. This initiative was done to call the attention of everyone, including health authorities, leaders, and communities to unite, act, and eradicate neglected tropical diseases (NTDs). According to the WHO, NTDs primarily affect the most vulnerable populations, where clean water availability, sanitation, and access to health care are inadequate in low- and middle-income countries of Africa, Asia, and Latin America. These pathologies affect over one billion people worldwide and are responsible for thousands of preventable deaths. Caused mostly by viruses, bacteria, parasites, fungi, and toxins, NTDs can blind, disable, and disfigure people. These diseases can also affect the ability of a person to stay in school, earn a living, or even be accepted by the community due to disease-related stigma.

The WHO has updated the list of NTDs to include leishmaniasis, malaria, sleeping sickness, filariasis, snakebite envenoming, and Chagas disease. In addition, emerging diseases such as dengue, chikungunya, and zika infections are also considered NTDs. Historically, NTDs have long been overlooked in the global health agenda, attracting little attention and low funding. Currently, there are few tools available to diagnose and treat those diseases. However, apart from the symbolism behind the World Neglected Tropical Diseases Day, research initiatives fighting NTDs have been conducted over the last years, paving the way for the development of new programs for prevention, diagnosis, and treatment of NTDs. A number of institutions and research groups have dedicated their notable work to investigating vaccines, diagnostics, and medicines to prevent, diagnose, and treat NTDs.

The field in which nanomaterials are used for diagnosing, monitoring, controlling, preventing, and treating diseases is called “nanomedicine” [1]. Potentially beneficial properties of nanomedicines include enhanced drug solubility, improved bioavailability, targeted drug delivery, longer half-life, and reduced toxicity. This thematic issue covers pre-clinical research employing chemotherapeutic or prophylactic nanomedicines against NTDs in a concise article collection. Among the articles, an interesting strategy to improve the bioavailability of benzonidazole towards Chagas disease has been presented by Muraca and colleagues, who reported a stable and safe nanostructured lipid formulation with potential effects against *Trypanosoma cruzi* [2]*.* In turn, Morilla and collaborators presented a critical review on nanomedicines and Chagas disease, highlighting the potential of oral nanocrystals and parenteral nano-immunostimulants to treat this NTD [3].

Moving to leishmaniases, Verçoza et al. evaluated the therapeutic potential of green superparamagnetic iron oxide nanoparticles (SPIONs) for treating cutaneous lesions caused by *Leishmania amazonensis.* The selectivity index for intracellular amastigotes was more than 240 times higher compared to that of current prescribed drugs to treat the disease, making SPIONs strong candidates for a new therapeutic approach against leishmaniasis [4]. Dourado and collaborators, who showed the therapeutic potential of curcumin-loaded nanocarriers, have also focused their review on these vector-borne NTDs [5].

With an emphasis on the treatment of schistosomiasis using nanoparticles, Carvalho and colleagues provided a comprehensive review on the field. Herein, the authors have accessed different databases, finding inorganic and polymeric nanoparticles as the most investigated nanosystems towards schistosomiasis, an acute and chronic parasitic NTD caused by blood-feeding nematodes of the genus *Schistosoma* [6].

Another important contribution to this thematic issue focused on development of nanoemulsions containing plant-based insecticides for vector control. In this work, Duarte and colleagues developed and characterized nanoemulsions encapsulating monoterpenes, which exhibited significant lethality against third-instar *Aedes aegypti* larvae [7]. This warrants further investigation on eco-friendly insecticides to fight *Aedes aegypti*, the primary vector of dengue, zika, and chikungunya.

Overall, this article collection was conceived to be an original literature resource converging nanomedicine and NTDs. All high-quality contributions emphasized the design and applications of nanomaterials as potential solutions for these diseases. We thank all the authors for submitting their articles. Meanwhile, we hope scientists, health authorities, and communities continue to fight against NTDs. And, who knows, maybe we will have a day to celebrate the cure or effective control of these diseases, promoting life quality for vulnerable populations.

Eder Lilia Romero, Katrien Van Bocxlaer and Fabio Rocha Formiga

Bernal, York and Recife, June 2024

## Data Availability

Data sharing is not applicable as no new data was generated or analyzed in this study.

## References

[1] Zhao Q, Cheng N, Sun X, Yan L, Li W (2023). Front Bioeng Biotechnol.

[2] Muraca G, Ruiz M E, Gambaro R C, Scioli-Montoto S, Sbaraglini M L, Padula G, Cisneros J S, Chain C Y, Álvarez V A, Huck-Iriart C (2023). Beilstein J Nanotechnol.

[3] Morilla M J, Ghosal K, Romero E L (2024). Beilstein J Nanotechnol.

[4] Verçoza B R F, Bernardo R R, de Oliveira L A S, Rodrigues J C F (2023). Beilstein J Nanotechnol.

[5] Dourado D, Silva Medeiros T, do Nascimento Alencar É, Matos Sales E, Formiga F R (2024). Beilstein J Nanotechnol.

[6] Carvalho L, Sarcinelli M, Patrício B (2024). Beilstein J Nanotechnol.

[7] Duarte J L, Di Filippo L D, de Faria Mota Oliveira A E M, Sábio R M, Marena G D, Bauab T M, Duque C, Corbel V, Chorilli M (2024). Beilstein J Nanotechnol.

